# Understanding ethnic diversity in open dementia neuroimaging data sets

**DOI:** 10.1093/braincomms/fcad308

**Published:** 2023-11-08

**Authors:** Nicholas Yew Wei Heng, Timothy Rittman

**Affiliations:** Department of Neurosciences, University of Cambridge, Herchel Smith building, Cambridge Biomedical Campus, Robinson Way, Cambridge CB2 0SZ, UK; Department of Neurosciences, University of Cambridge, Herchel Smith building, Cambridge Biomedical Campus, Robinson Way, Cambridge CB2 0SZ, UK

**Keywords:** neuroimaging, ethnicity, neurodegenerative disorders, dementia

## Abstract

Ethnic differences in dementia are increasingly recognized in epidemiological measures and diagnostic biomarkers. Nonetheless, ethnic diversity remains limited in many study populations. Here, we provide insights into ethnic diversity in open-access neuroimaging dementia data sets. Data sets comprising dementia populations with available data on ethnicity were included. Statistical analyses of sample and effect sizes were based on the *Cochrane Handbook*. Nineteen databases were included, with 17 studies of healthy groups or a combination of diagnostic groups if breakdown was unavailable and 12 of mild cognitive impairment and dementia groups. Combining all studies on dementia patients, the largest ethnic group was Caucasian (20 547 participants), with the next most common being Afro-Caribbean (1958), followed by Asian (1211). The smallest effect size detectable within the Caucasian group was 0.03, compared to Afro-Caribbean (0.1) and Asian (0.13). Our findings quantify the lack of ethnic diversity in openly available dementia data sets. More representative data would facilitate the development and validation of biomarkers relevant across ethnicities.

## Teaser

Heng and Rittman highlight a lack of ethnic diversity and disproportionately larger Caucasian populations in open dementia data sets, despite increasing recognition of the impact of ethnic differences in epidemiological measures and diagnostic biomarkers. It is hoped that this will help to raise awareness and broaden inclusion within the field.

## Graphical Abstract

**Graphical Abstract:**
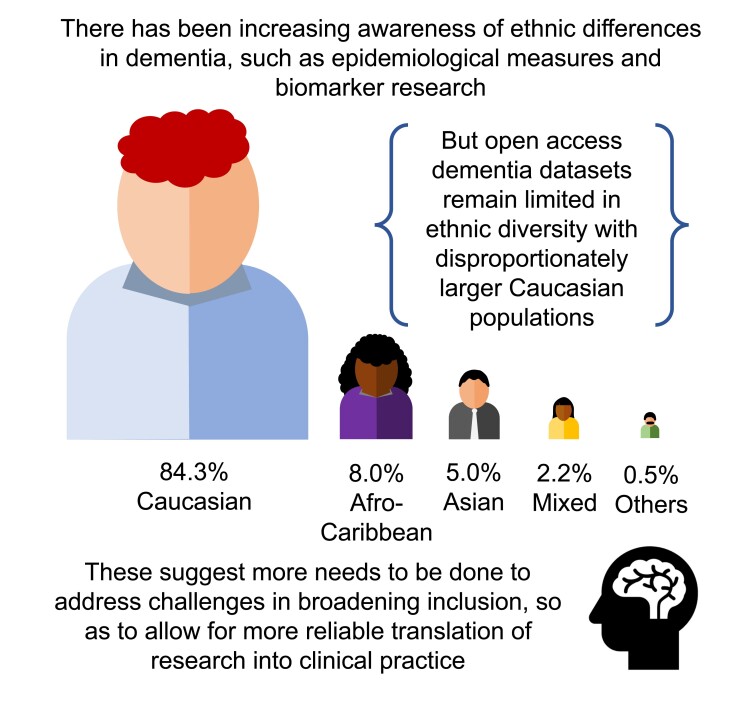

## Introduction

The past few decades have seen growing interest in the field of biomarkers for neurodegenerative conditions. The neuroimaging community has led the way in open data,^1^ facilitating an explosion of research in neuroimaging biomarkers for dementia.^2^ This interest is in the context of an increasing global burden of neurodegenerative disorders, particularly in relation to the impact of Alzheimer’s disease and other dementias on an increasingly aging population.^3^ Crucially, it has been estimated that the prevalence of dementia will increase from 57.4 million cases globally in 2019 to 152.8 million cases in 2050,^4^ posing a considerable risk to global healthcare and society in the near future. There has been emerging evidence of ethnic differences amongst dementia populations, not only in incidence^5,6^ but also in CSF and imaging biomarkers.^7,8^

Nonetheless, many studies remain homogenous in the ethnicity of participants.^9^ This may hinder the translation of results to real-world applications. As such, we aimed to provide insights into the ethnic diversity of currently available open neuroimaging dementia databases worldwide.

## Materials and methods

**Figure 1:**
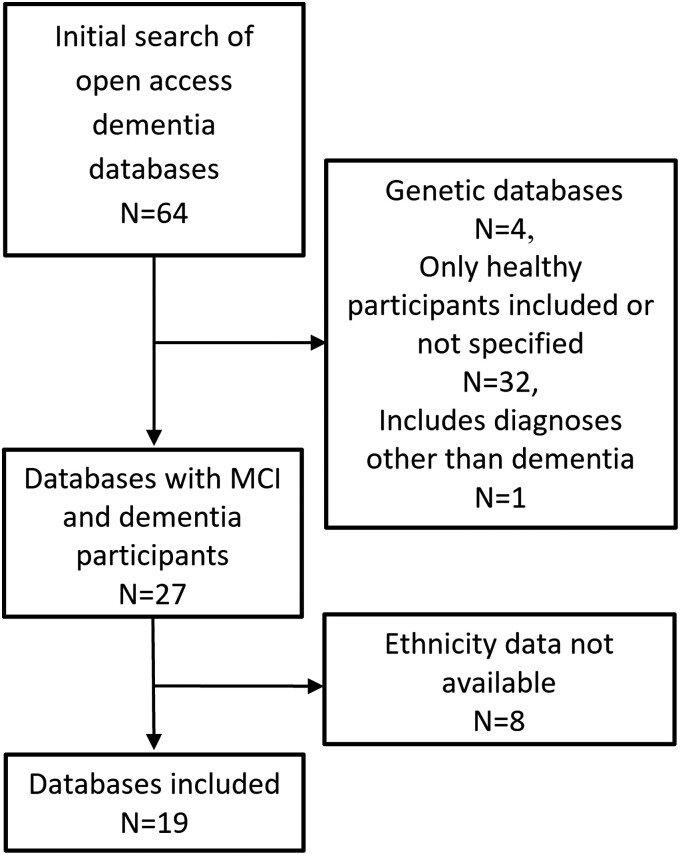
Flowchart depicting selection and inclusion of open-access dementia databases.

We compiled and analysed demographic data reported by open-access dementia databases. Databases were included if they consisted of (i) patients with a diagnosis of dementia or mild cognitive impairment (MCI) and (ii) demographic data including the breakdown of ethnicities. Data sets were identified through online research platforms including the Global Alzheimer’s Association Interactive Network (GAAIN, https://www.gaain.org/), individual database repositories and via peer-reviewed journal articles. We excluded data sets of solely genetic forms of dementia since these may be associated with specific ethnicities or include large families that might bias the estimation of the distribution of ethnicities. A total of 64 databases were found, but 45 were subsequently excluded as they only included healthy controls, included other diagnoses, consisted only of genetic forms of dementia or had no available data on demographics (Fig. 1).

Given the different definitions of ethnicities available, we took a pragmatic approach using the most widely used terms in the literature that permitted comparison between studies.

Statistical analyses on combined mean and standard deviation were performed as laid out by the *Cochrane Handbook*,^10^ and effect size calculations were done using the pwr package in R (version 4.2.2).^11^ To compare samples of presumed equal sizes, we performed a power calculation for a two-sample *t*-test, estimating the effect size or sample size detectable with 90% power at a significance level (*P*-value) of 0.05. Sample sizes were initially computed by setting a range of effect sizes, while minimum detectable effect sizes for single ethnic groups were then calculated using the aggregated dementia patient populations of different ethnicities from the open-access dementia databases.

## Results

### Demographics of dementia databases

A total of 19 dementia neuroimaging data sets were included, separated into three diagnostic groups, with 17 including healthy participants or reflecting total number of participants if breakdown of diagnostic groups was not available (Supplementary Table 1), 12 including patients with MCI (Supplementary Table 2) and 12 including patients with dementia (Table 1).^12-27^ In these tables, two entries for the Alzheimer’s Disease Neuroimaging Initiative (ADNI) data set were made due to the separation of the ADNI-1 from ADNIGO and ADNI-2 cohorts. The majority of patients were from North America and Europe, with the two largest databases being from the National Alzheimer’s Coordinating Center (NACC) and UK Biobank, respectively, in which there were a considerably higher percentage of Caucasians compared to other ethnicities.

**Table 1:** Breakdown of demographic data of patients with dementia in databases globally as separated by region

S/N	Database	Number of patients with dementia	Mean age (SD)	Gender		Ethnicity				
				Male (%)	Female (%)	Caucasian (%)	Afro-Caribbean (%)	Asian (%)	Mixed (%)	Others (%)
**North America**										
**1**	ADNI-1 (USA, 12)	192	75.3 (7.5)	52.6	47.4	92.2	4.2	1.0		2.1% as Hispanic, 0.5% others
**2**	ADNIGO and ADNI-2 (USA, 13)	145	74.6 (8.1)	59.0	41.0	91.0	4.1	3.5	1.4	
**3**	NACC (USA, 14)	20 053	75.9 (10.8)	48.0	52.0	83.3	9.2	2.2	2.6	0.5% American Indian, 0.1% Hawaiian/Pacific Islander, 2.1% others
**4**	HABLE (USA, 15)	185	68.2 (9.9)	45.9	54.1	71.4	28.6			
**South America**										
**5**	Argentina-ADNI (Argentina, 20)	12	77.9 (5.5)	41.7	58.3	100.0				
**Europe**										
**6**	I-ADNI (Italy, 21)	201	71.8 (8.4)	38.8	61.2	100.0				
**7**	UK Biobank (UK, 22)	2778	64.7 (4.2)	45.3	54.7	95.5	1.4	1.4	0.3	0.4% others
**8**	ARWIBO (Italy, 23)	402	73.5 (8.5)	36.8	63.2	100.0				
**9**	EDSD (Italy, Germany, Netherlands, 24)	139	73.0 (8.0)	43.2	56.8	100.0				
**Asia**										
**10**	J-ADNI (Japan, 25)	149	73.7 (6.6)	43.0	57.0			100.0		
**11**	WMH-AD (Taiwan, from GAAIN)	43	77.2 (7.7)	25.6	74.4			100.0		
**12**	KBASE (South Korea, 26)	87	73.0 (8.1)	31.0	69.0			100.0		
**13**	DART (Taiwan, from GAAIN)	435						100.0		
**Total**	(Excluding DART due to lack of data)	24 821	74.4 (10.7)							

### Effect size analyses

To understand how the breakdown of ethnicity in these data sets could affect research studies, we calculated the sample sizes required for a range of effect sizes. For example, based on a recent systematic review and meta-analysis on fluid biomarkers for Alzheimer’s disease,^8^ it was found that CSF p-tau_181_ and t-tau levels were significantly higher in the Caucasian population compared to African Americans with MCI, with a standard mean difference of −0.50 [95% confidence interval (CI) −0.73 to −0.28] and −0.52 (95% CI −0.75 to −0.30), respectively—though bearing in mind that these did not necessarily inform the effect size in other biomarkers or ethnicities. Therefore, using an estimated effect size of 0.50 and basing off a power calculation of 90% and significance level of 0.05, the number of patients required to detect a difference was *n* = 86 each for two groups of patients of different ethnicities. We went on to calculate sample sizes for a range of effect sizes to obtain a better idea of the sample size to consider when planning future studies. In addition, we assessed whether the available data were sufficient to make comparisons between the Caucasian population and other ethnic groups (Table 2).

**Table 2:** Sample sizes required for specific effect sizes to be obtained based on power calculations of 90% and significance level of 0.05, with subsequent columns showing whether comparisons between ethnic groups can be performed based on currently available data

Effect size	Sample size required	Caucasian versus Afro-Caribbean	Caucasian versus Asian	Caucasian versus mixed	Caucasian versus others
0.5	86	✓	✓	✓	✓
0.3	235	✓	✓	✓	✘
0.2	527	✓	✓	✘	✘
0.1	2103	✘	✘	✘	✘
0.05	8407	✘	✘	✘	✘
0.01	210 150	✘	✘	✘	✘

In an alternate approach, using available data for patients with dementia in those data sets combined with similar power calculation of 90% and significance of 0.05, we determined the smallest detectable effect size given currently available data (Table 3). The Caucasian population had the smallest minimum detectable effect size at 0.03 due to its size.

**Table 3:** Smallest effect sizes detected using population sizes based on available ethnicity data from dementia databases with similar parameters of 90% power and significance level of 0.05

Ethnicity group	Total number of patients with dementia	Smallest effect size detected
Caucasian	20 547	0.03
Afro-Caribbean	1958	0.10
Asian	1211	0.13
Mixed	525	0.20
Others	134	0.40

## Discussion

With the increasing number of studies focusing on ethnic differences in dementia, there is little doubt that more emphasis needs to be placed on the role that ethnic differences play in biomarker research. Our findings suggest that despite the vast amount of comprehensive and high-quality data available worldwide, most participants come from a Caucasian background, limiting comparison to other populations. Considerable numbers of patients are required for assessing small magnitude effect sizes—which becomes particularly important when trying to identify potentially subtle differences within or between ethnic groups. The minimum detectable effect size can therefore act as a guide or threshold towards that end. In fact, the majority of the population sizes were made up of two large databases in the UK and the USA. We hope these findings can act as a starting point into deciding how to expand representation of different ethnic groups in future studies on dementia.

Understanding the limitations of currently available data can provide an invaluable opportunity to uncover and tackle the challenges to ensuring ethnic diversity in studies. Firstly, there needs to be a focus on expanding access and improving communication with underserved populations through addressing barriers to communication, such as via provision of dual-language instructional materials or translators,^28,29^ and forging and empowering stronger patient and public involvement through consultations and collaborations.^30^ In the drive to broaden recruitment strategies, consideration also needs to be given to adequate financial compensation to improve accessibility.^29^ Furthermore, within institutions themselves, there should be an ongoing push to enact training on bias and advocate for guidelines focused on fairness and generalizability in research,^31^ such as those from the Committee on Best Practice in Data Analysis and Sharing.^32^ These approaches may begin to address the mistrust of scientific communities that has been identified in underserved populations due to past unethical research and serve to better facilitate participation, understanding and awareness.^29^

In addition, we were only able to obtain data for openly available data sets. We know from published data and from personal contacts that many studies around the world use local cohorts, and some large national cohorts are not shared with the wider community. We advocate exploring the barriers to sharing those data, including the concerns of those who have collected and curated those data sets.

There are several limitations to our study—the first being that we were unable to comment on the representativeness (as opposed to heterogeneity) of the combined characteristics of the populations included in the database. Data on global ethnicity are not readily available, and classifications differ between different countries, making it difficult to draw comparisons. Nonetheless, with databases mostly consisting of participants within the Western Hemisphere, comparisons between their ethnicity breakdown and the 2021 census data for the USA^33^ and the UK—England and Wales^34^ (Table 4), suggest a disproportionately larger Caucasian population included in these databases than in the general population. Secondly, a considerable number of studies were excluded due to the lack of available demographic data, and those that were included were mainly based in the Western Hemisphere, which may mean we have underestimated the non-Caucasian ethnicities actually available.

**Table 4:** The 2021 census data for the United States of America (USA) and the United Kingdom (UK)—England and Wales, alongside the ethnicitybreakdown of the two largest data sets consisting of patients with dementia, namely National Alzheimer's Coordinating Center (NACC) (USA) and theUnited Kingdom Biobank (UK)

Ethnicity group	USA 2021 census data, %	UK 2021 census data for England and Wales, %	NACC, %	UK Biobank, %
Caucasian	75.7	81.7	83.3	95.5
Afro-Caribbean	13.6	4.0	9.2	1.4
Asian	6.3	9.3	2.2	1.4
Mixed	3.0	2.9	2.6	0.3
Others	1.6	2.1	2.7	0.4

In our analysis, we assume that data sets can easily be combined. In fact, harmonization between data sets presents a significant methodological challenge given that protocols differ and site effects need to be modelled.^35,36^ This is particularly a challenge for combining neuroimaging data despite the increasing availability of tools for this purpose such as ComBat.^37^

## Conclusion

With increasing awareness of the differences between ethnicities in dementia, it is imperative that we begin to prioritize and broaden biomarker research to better understand underlying mechanisms, to address the challenges associated with ethnic diversity in studies and ultimately to pave the way for reliable translation into clinical practice.

## Supplementary Material

fcad308_Supplementary_Data

## Data Availability

Data sharing is not applicable to this article as no new data were created in this study.
