# Supplementary material for: Understanding ethnic diversity in open dementia neuroimaging data sets
Source: Brain Commun. 2023 Nov 8;5(6):fcad308. doi: 10.1093/braincomms/fcad308 (PMC10667030; doi:10.1093/braincomms/fcad308)
Supplement: fcad308_Supplementary_Data [file fcad308_supplementary_data.pdf]

Supplementary Tables

Supplementary Table 1. Table showing the breakdown of demographics data of healthy controls or total numbers in databases globally as separated by region.

S/N	Database	Number of healthy controls or total numbers	Mean age (SD)	Gender		Ethnicity				
				Male (%)	Female (%)	Caucasian (%)	Afro-Caribbean (%)	Asian (%)	Mixed (%)	Others (%)
**North America**										
**1**	ADNI-1 (USA) (1)	229	75.8 (5.0)	52.0	48.0	90.8	7.0	1.3	-	0.9% Hispanic
**2**	ADNIGO and ADNI-2 (USA) (2)	287	73.0 (6.1)	46.0	54.0	90.2	5.9	1.7	1.7	0.3% American-Indian/Alaskan
**3**	NACC (USA) (3)	16,825	72.8 (11.4)	35.0	65.0	76.8	14.8	3.1	3.4	0.6% American-Indian, 0.1% Hawaiian/Pacific Islander, 1.2% others
**4**	HABLE (USA) (4)	2032	65.0 (8.4)	34.9	65.1	84.0	15.9	-	-	N=1 mixed
**5**	OASIS-3 (USA) (5)	1098	68.8 (range 42.5-95.6)	44.4	55.6	84.3	15.3	0.4	-	-
**6**	CPAD (USA) (From GAAIN and (6))	6500 (Total)	-	43.8	56.2	91.4	2.5	4.7	0.05	0.2% American-Indian/Alaskan, 0.03% Hawaiian/Pacific, 1.0% others, 0.09% unknown (N=6)
**7**	CLSA (Canada) (From GAAIN and (7))	51,214 (Total)	-	49.0	50.9 (0.03 unknown)	95.1	0.7	1.8	1.1	0.5% American-Indian/Alaskan, 0.8% Hispanic, 0.8% unknown
**8**	TARCC (USA) (From GAAIN and (8))	3670 (Total)	-	38.3	61.7	93.0	4.1	2.6	-	0.2% American-Indian/Alaskan, 0.05% Hawaiian/Pacific, 0.05% unknown (N=2)
**South America**										
**9**	Argentina-ADNI (Argentina) (9)	14	70.1 (8.2)	28.6	71.4	100.0	-	-	-	-
**Europe**										

**10**	I-ADNI (Italy) (10)	7	70.0 (10.5)	57.1	42.9	100.0	-	-	-	-
**11**	UK Biobank (UK) (11)	493,735	56.4 (8.1)	44.3	52.9	93.9	1.6	2.3	0.5	0.9% others
**12**	ARWIBO (Italy) (12)	1482	51.6 (15.4)	39.1	60.9	100.0	-	-	-	-
**13**	EDSD (Italy, Germany, Netherlands) (13)	194	69.0 (6.0)	49.0	51.0	100.0	-	-	-	-
**Asia**										
**14**	J-ADNI (Japan) (14)	154	68.3 (5.8)	48.1	51.9	-	-	100.0	-	-
**15**	WMH-AD (Taiwan) (From GAAIN)	19	70.4 (8.2)	68.4	31.6	-	-	100.0	-	-
**16**	KBASE (South Korea) (15)	365 (Total)	62.8 (15.2)	48.5	51.5	-	-	100.0	-	-
**17**	DART (Taiwan) (From GAAIN)	938 (Total)	-	43.8	56.2	-	-	100.0	-	-
**18**	AMED (Japan) (From GAAIN)	38 (Total)	-	60.5	39.5	-	-	100.0	-	-
**Total**	(Excluding OASIS-3 due to range, and CLSA, TARCC, CPAD, DART and AMED due to lack of data)	578,801	57.0 (8.8)							

Supplementary Table 2. Table showing the breakdown of demographics data of patients with mild cognitive impairment (MCI) in databases globally as separated by region.

S/N	Database	Number of patients with MCI	Mean age (SD)	Gender		Ethnicity				
				Male	Female	Caucasian	Afro-Caribbean	Asian	Mixed	Others
**North America**										
**1**	ADNI-1 (USA) (1)	398	74.7 (7.4)	64.6%	35.4%	90.5%	3.5%	2.3%	-	3.5% classed as Hispanic, 0.3% as others
**2**	ADNIGO and ADNI-2 (USA) (2)	461	71.6 (7.4)	55.3%	44.7%	93.3%	2.8%	1.1%	1.5%	0.2% American-Indian/Alaskan, 0.4% Hawaiian/Pacific Islander, 0.7% others
**3**	NACC (USA) (3)	8404	75.7 (10.2)	47.0%	53.0%	75.6%	15.4%	3.0%	3.5%	0.7% American-Indian, 0.1% Hawaiian/Pacific Islander, 1.6% others
**4**	HABLE (USA) (4)	490	65.9 (9.0)	44.9%	55.1%	70.6%	29.4%	-	-	-
**South America**										
**5**	Argentina-ADNI (Argentina) (9)	23	74.0 (7.4)	47.9%	52.1%	100.0%	-	-	-	-
**Europe**										
**6**	PharmaCog (Europe) (16)	151	69.3 (7.4)	43.7%	56.3%	100.0%	-	-	-	-
**7**	I-ADNI (Italy) (10)	54	69.5 (7.2)	50.0%	50.0%	100.0%	-	-	-	-
**8**	ARWIBO (Italy) (12)	308	71.2 (8.0)	38.3%	61.7%	100.0%	-	-	-	-
**9**	EDSD (Italy, Germany, Netherlands) (13)	160	73.0 (7.0)	56.9%	43.1%	100.0%	-	-	-	-
**10**	DCL (Spain) (From GAAIN and investigators)	308	75.1 (8.7)	51.3%	48.7%	100.0%	-	-	-	-
**Asia**										
**11**	J-ADNI (Japan) (14)	234	73.0 (5.9)	49.6%	50.4%	-	-	100.0%	-	-
**12**	WMH-AD (Taiwan) (From GAAIN)	27	73.4 (7.7)	48.1%	51.9%	-	-	100.0%	-	-
**13**	KBASE (South Korea) (15)	139	73.7 (7.0)	33.8%	66.2%	-	-	100.0%	-	-
**Total**		11,157	74.7 (9.9)							

References for supplementary material

1. Petersen RC, Aisen PS, Beckett LA, Donohue MC, Gamst AC, Harvey DJ, Jack CR, Jagust WJ, Shaw LM, Toga AW, Trojanowski JQ, Weiner MW. Alzheimer's Disease Neuroimaging Initiative (ADNI). Neurology. 2010; 74(3): 201-209.

2. Aisen PS, Petersen RC, Donohue M, Weiner MW. ADNI 2 Clinical Core: Progress and Plans. Alzheimers Dement. 2015; 11(7): 734-739.

3. The NIA Alzheimer's Disease Research Centers Program. National Alzheimer's Coordinating Center. 2023. Available from: https://naccdata.org/requesting-data/data-summary/uds. Accessed July 30 2023.

4. O'Bryant SE, Johnson LA, Barber RC, Braskie MN, Christian B, Hall JR, Hazra N, King K, Kothapalli D, Large S, Mason D, Matsiyevskiy E, McColl R, Nandy R, Palmer R, Petersen M, Philips N, Rissman RA, Shi Y, Toga AW, Vintimilla R, Vig R, Zhang F, Yaffe K; HABLE Study Team. The Health & Aging Brain among Latino Elders (HABLE) study methods and participant characteristics. Alzheimers Dement (Amst). 2021;13(1):e12202.

5. LaMontague PJ, Benzinger TLS, Morris JC, Keefe S, Hornbeck R, Xiong C, Grant E, Hassenstab J, Moulder K, Vlassenko AG, Raichile ME, Cruchaga C, Marcus D. OASIS-3: Longitudinal Neuroimaging, Clinical, and Cognitive Dataset for Normal Aging and Alzheimer Disease. 2019. medRxiv. doi: 10.1101/2019.12.13.19014902.

6. Critical Path for Alzheimer's Disease. Critical Path Institute 2023. Available from: https://c-path.org/programs/cpad/tools-and-teams/cpad-codr/#:~:text=It%20is%20openly%20available%20to,drug%20candidates%20from%20sponsor%20companies. Accessed July 30 2023.

7. Raina P, Wolfson C, Kirkland S, Griffith LE, Balion C, Cossette B, Dionne I, Hofer S, Hogan D, van den Heuvel ER, Liu-Ambrose T, Menec V, Mugford G, Patterson C, Payette H, Richards B, Shannon H, Sheets D, Taler V, Thompson M, Tuokko H, Wister A, Wu C, Young L. Cohort Profile: The Canadian Longitudinal Study on Aging (CLSA). Int J Epidemiol. 2019 Dec 1;48(6):1752-1753j. doi: 10.1093/ije/dyz173. Erratum in: Int J Epidemiol. 2019 Dec 1;48(6):2066.

8. The Texas Alzheimer's Research and Core Consortium. Darrell K Royal Texas Alzheimer's Initiative 2016. Available from: http://www.txalzresearch.org/research/the-texas-harris-alzheimers-research-study/. Accessed July 30 2023.

9. Méndez PC, Calandri I, Nahas F, Russo MJ, Demey I, Martín ME, Clarens MF, Harris P, Tapajoz F, Campos J, Surace EI, Martinetto H, Ventrice F, Cohen G, Vázquez S, Romero C, Guinjoan S, Allegri RF, Sevlever G. Argentina-Alzheimer's disease neuroimaging initiative (Arg-ADNI): neuropsychological evolution profile after one-year follow up. Arq Neuropsiquiatr. 2018;76(4):231-240.

10. Cavedo E, Redolfi A, Angeloni F, Babiloni C, Lizio R, Chiapparini L, Bruzzone MG, Aquino D, Sabatini U, Alesiani M, Cherubini A, Salvatore E, Soricelli A, Vernieri F, Scrascia F, Sinforiani E, Chiarati P, Bastianello S, Montella P, Corbo D, Tedeschi G, Marino S, Baglieri A, De Salvo S, Carducci F, Quattrocchi CC, Cobelli M, Frisoni GB. The Italian Alzheimer's Disease Neuroimaging Initiative (I-ADNI): validation of structural MR imaging. J Alzheimers Dis. 2014;40(4):941-52.

11. Swaddiwudhipong N, Whiteside DJ, Hezemans FH, Street D, Rowe JB, Rittman T. Pre-diagnostic cognitive and functional impairment in multiple sporadic neurodegenerative diseases. Alzheimers Dement. 2022.

12. NeuGRID2 consortium. NeuGRID. 2012. Available from: https://www.neugrid2.eu/index.php/introduction/. Accessed July 30 2023.

13. Brueggen K, Grothe MJ, Dyrba M, Fellgiebel A, Fischer F, Filippi M, Agosta F, Nestor P, Meisenzahl E, Blautzik J, Frölich L, Hausner L, Bokde ALW, Frisoni G, Pievani M, Klöppel S, Prvulovic D, Barkhof F, Pouwels PJW, Schröder J, Hampel H, Hauenstein K, Teipel S. The European DTI Study on Dementia - A multicenter DTI and MRI study on Alzheimer's disease and Mild Cognitive Impairment. Neuroimage. 2017;144(Pt B):305-308.

14. NBDC Human Database. NBDC Research ID: hum0043.v1. 2016. https://humandbs.biosciencedbc.jp/en/hum0043-v1. Accessed July 30 2023.

15. Byun MS, Yi D, Lee JH, Choe YM, Sohn BK, Lee JY, Choi HJ, Baek H, Kim YK, Lee YS, Sohn CH, Mook-Jung I, Choi M, Lee YJ, Lee DW, Ryu SH, Kim SG, Kim JW, Woo JI, Lee DY; KBASE Research Group. Korean Brain Aging Study for the Early Diagnosis and Prediction of Alzheimer's Disease: Methodology and Baseline Sample Characteristics. Psychiatry Investig. 2017;14(6):851-863.

16. Albani D, Marizzoni M, Ferrari C, Fusco F, Boeri L, Raimondi I, Jovicich J, Babiloni C, Soricelli A, Lizio R, Galluzzi S, Cavaliere L, Didic M, Schönknecht P, Molinuevo JL, Nobili F, Parnetti L, Payoux P, Bocchio L, Salvatore M, Rossini PM, Tsolaki M, Visser PJ, Richardson JC, Wiltfang J, Bordet R, Blin O, Forloni G, Frisoni GB; PharmaCog Consortium. Plasma Aβ42 as a Biomarker of Prodromal Alzheimer's Disease Progression in Patients with Amnestic Mild Cognitive Impairment: Evidence from the PharmaCog/E-ADNI Study. J Alzheimers Dis. 2019;69(1):37-48. doi: 10.3233/JAD-180321. PMID: 30149449.
